# Alteration of Gut Microbiota in Patients With Epilepsy and the Potential Index as a Biomarker

**DOI:** 10.3389/fmicb.2020.517797

**Published:** 2020-09-18

**Authors:** Xue Gong, Xu Liu, Chu Chen, Jingfang Lin, Aiqing Li, Kundian Guo, Dongmei An, Dong Zhou, Zhen Hong

**Affiliations:** Department of Neurology, West China Hospital, Sichuan University, Chengdu, China

**Keywords:** epilepsy, human, fecal microbiota, 16S RNA sequence, ROC

## Abstract

**Objective:**

To explore the structure and composition of the fecal microbiota of patients with epilepsy.

**Methods:**

Variations in the fecal microbiota between patients with epilepsy and healthy controls (HCs) from the same household were investigated and validated by utilizing 16S ribosomal RNA sequencing in two independent cohorts [exploration cohort (*N* = 55 patients and *N* = 46 HCs) and validation cohort (*N* = 13 patients and *N* = 10 HCs)].

**Results:**

The alpha diversity indexes of the specimens from patients with epilepsy were much lower than those from the HCs (*p* < 0.05). The structure and composition of the fecal microbiota differed between patients with different clinical prognoses and between patients and HCs (Adonis: *p* < 0.05). Microbiome alterations in patients with epilepsy included increases in *Actinobacteria* and *Verrucomicrobia* and decreases in *Proteobacteria* at the phylum level and increases in *Prevotella_9*, *Blautia*, *Bifidobacterium*, and others at the genus level [linear discriminant analysis (LDA): 3.5] Patients with drug-resistant epilepsy showed enrichment of bacterial taxa in *Actinobacteria*, *Verrucomicrobia*, and *Nitrospirae* and the genera *Blautia*, *Bifidobacterium*, *Subdoligranulum*, *Dialister*, and *Anaerostipes* (Kruskal-Wallis test: *p* < 0.05). Analysis of gut microbiome indicated predictive ability for disease diagnosis, with an area under the receiver operating characteristic (ROC) curve (AUC) of 0.97 (95% CI, 0.84–0.98). Applying the model to our validation cohort resulted in an AUC of 0.96 (95% CI, 0.75–0.97). Notably, the model could distinguish drug-resistant from drug-sensitive epilepsy (AUC = 0.85, 95% CI: 0.69–0.94).

**Conclusion:**

Patients with epilepsy exhibit substantial alterations of fecal microbiota composition, and specific gut commensal strains are altered depending on different clinical phenotypes and thus could serve as potential biomarkers for disease diagnosis.

## Introduction

Epilepsy is a common and devastating neurological disorder featured by an enduring predisposition to generate epileptic seizures. The condition is associated with psychological, cognitive, and social consequences (Fisher Robert et al., 2014; Devinsky et al., 2018). It affects 65 million people worldwide and is the third leading contributor to the global burden of neurological disorders (Ngugi et al., 2010; Devinsky et al., 2018). The mechanism of epileptogenesis is multifactorial, and the etiology of many patients is still unknown.

More than 85% of patients with epilepsy live in poverty-stricken areas (Kurupath, 2009) where sanitation is poor. Moreover, cases of epilepsy are reported more in low-income countries than in high-income countries (Fiest Kirsten et al., 2017; Devinsky et al., 2018). Accumulating evidence indicated that environmental factors have significant influence on gut microbiota composition (Dahlin and Prast-Nielsen, 2019). Also, numerous clinical studies have revealed that antibiotic use can enhance the risk of developing symptomatic seizures or status epilepticus in patients with epilepsy (Sutter et al., 2015; Olson et al., 2018b). A low-carbohydrate, high-fat ketogenic diet (KD) is an effective therapy for drug refractory epilepsy (Kwan and Brodie, 2000; Olson et al., 2018b). According to a recent study in mice, gut microbiota is necessary and sufficient for seizure protection and impacts the antiseizure effects of a KD (Olson et al., 2018a). All of the above results imply a possible role of the microbiota in mitigating seizure likelihood (Olson et al., 2018b). The intestinal microbiota-brain tract, which is the largest axis has been recently shown to sense and react to dynamic ecosystem changes by converting pathogen-associated chemical cues from the environment into neuronal impulses (Yoo and Mazmanian, 2017), thus implicating a potential role of the gut microbiota in epileptogenesis.

To date, very few studies have assessed the role of alteration in the gut microbiota in epilepsy (Dahlin and Prast-Nielsen, 2019; Lum et al., 2019; Safak et al., 2019). The authors of these studies compared the taxa between drug-sensitive epilepsy (DSE) and drug-resistant epilepsy (DRE) groups by using 16S rRNA gene sequencing without enrollment of healthy controls (HCs) or controlling some the critical factors to the gut microbiota, such as diet and the environment (Peng et al., 2018). Thus far, the variations in the gut microbiota in healthy individuals versus individuals with the disease is poorly understood, making clarification of the possible function of the gut microbiota in epileptogenesis mechanisms necessary. Here, for the first time, we conducted a detailed assessment of the fecal microbiota in patients with epilepsy with unknown etiology and HCs from the same household. Subgroup analyses were also conducted by stratifying patients with different clinical phenotypes. Our data further revealed that the identified microbial signature can act as a valuable tool for disease diagnosis prediction.

## Materials and Methods

### Study Cohort and Study Design

Two independent cross-sectional analyses were performed, including exploration and validation cohorts, the division of which was performed prior to data analysis. To explore the alteration of the microbiota between the two groups and between subgroups of patients with epilepsy, 55 patients with epilepsy and 46 HCs from the same household (exploration cohort) were consecutively enrolled between July 1, 2016, and May 1, 2017, from the inpatient department of neurology center of West China Hospital. Then, to validate the potential prediction effect for epilepsy, an independent cohort composed of 13 patients and 10 controls (validation cohort) was enrolled between March 1, 2018, and July 1, 2018, from the inpatient department of NWCH. All subjects were subjected to extensive clinical assessments that involved medical history, neurological and physical examinations, laboratory tests, and neuropsychological evaluations. The participants were made aware of the aim of the study and were allowed to undergo tests and fill the questionnaires voluntarily. Thus, all patients provided written informed consent. The approval for this study was provided by the Research Ethics Committee of the Medical School of Sichuan University.

The inclusion criteria for normal controls and patients with epilepsy were as follows:

HCs: bacterial communities in the gut tend to be more similar in couples compared to people who are not living in the same households (Song et al., 2013). As such, we included healthy spouses as controls in both the exploration and validation cohorts in our study. Only couples who have lived together and shared a similar diet for at least 10 years were included.

Patients with epilepsy: (1) Patients definitively met the diagnosis criteria of epilepsy with unknown causes in accordance with the International League Against Epilepsy Classification framework in 2017 for at least 3 years (Fisher Robert et al., 2017), briefly: lacking localized MRI lesions, no known genetic metabolic disorders or severe systemic illnesses, and no family history of convulsion; and (2) patients ages ranged from 15 to 60 years.

The exclusion criteria for both patients with epilepsy and HCs were as follows: (1) antibiotics, probiotics, prebiotics, or synbiotics therapy in the last 3 months; (2) gastroenteritis history in the past 3 months; and (3) history of other autoimmune diseases (multiple sclerosis, optic neuromyelitis, systemic lupus erythematosus, rheumatoid arthritis, type 1 diabetes, and others); (4) history of bowel surgery; (5) pregnant or lactating; (6) history of neurologic or psychiatric disorder (Parkinson’s disease, Alzheimer’s disease, anxiety disorder, depression, autism spectrum disorder, schizophrenia, etc.); (7) history of additional regimen intake except for antiepileptic drugs (AEDs) within 6 months (vitamins, protein, unsaturated fatty acids, etc.); and (8) severe malnutrition or infection or drug or alcohol addiction.

In subsample analyses, we further grouped patients on the basis of DRE and DSE. DRE was defined as the failure of adequate trials of two or more tolerated, appropriately chosen and used AED regimens (whether administered as monotherapies or in combination) to achieve seizure freedom (Kwan et al., 2010).

### Clinical Information Collection

The demographics, disease duration, clinical manifestations, age of onset, results of auxiliary examinations and methods of treatment of these patients were retrieved from clinical review and/or medical records. In our previous study, to further control for diet, the participants were asked to fill a validated food frequency questionnaire (CHNS2015-FFQ). Data relating to 27 nutrients, which included carbohydrates, protein, and fat were retrieved from a nutritional database^1^.

### Fecal Collection and Quality Control

All participants were provided with sterile collection containers for sample collection (each tube was able to accommodate at least 50 g). All participants exhibited normal bowel habits and stool conditions during the examination period. Fecal core samples were all freshly collected from patients in the morning when the patients had an empty stomach, and were delivered to West China Hospital not more than 2 h after collection. Upon delivery, the samples were immediately preserved at −80°C, awaiting subsequent experiments which included DNA isolation and other assays.

### DNA Extraction and PCR Amplification

Microbial community genomic DNA was extracted from fecal samples using an E.Z.N.A.^®^ Soil DNA Kit (Omega Biotek, Norcross, GA, United States) according to the manufacturer’s instructions. The DNA extract was checked on a 1% agarose gel, and the DNA concentration and purity were determined with a NanoDrop 2000 UV-Vis spectrophotometer (Thermo Fisher Scientific, Wilmington, United States). The V3-V4 hypervariable region of the bacterial 16S rRNA gene was amplified with the primer pair 338F (5′-ACTCCTACGGGAGGCAGCAG-3′) and 806R (5′-GGACTACHVGGGTWTCTAAT-3′) by an ABI GeneAmp^®^ 9700 PCR thermocycler (ABI, CA, United States), with an eight-base sequence barcode unique to each sample at the 5’ end of 338F and 806R (Tang et al., 2020). PCR amplification of the 16S rRNA gene was performed as follows: initial denaturation at 95°C for 3 min, followed by 27 cycles of denaturation at 95°C for 30 s, annealing at 55°C for 30 s and extension at 72°C for 45 s; a single extension at 72°C for 10 min; and a hold at 4°C. The PCR mixtures contained 5 × TransStart FastPfu buffer (4 μL), 2.5 mM dNTPs (2 μL), 5 μM forward primer (0.8 μL), 5 μM reverse primer (0.8 μL), TransStart FastPfu DNA Polymerase (0.4 μL), template DNA (10 ng), and ddH2O (up to 20 μL). The PCR product was extracted from a 2% agarose gel and purified using an AxyPrep DNA Gel Extraction Kit (Axygen Biosciences, Union City, CA, United States) according to the manufacturer’s instructions and quantified using a QuantiFluor^TM^ ST fluorometer (Promega, United States). Targeted identification and measurement of fecal metabolomics were performed based on a validated method (Zhao et al., 2017).

### Illumina MiSeq Sequencing

Purified amplicons were pooled in equimolar amounts and paired-end sequenced (2 × 300) on an Illumina MiSeq platform (Illumina, San Diego, United States) according to standard protocols by Majorbio Bio-Pharm Technology Co., Ltd. (Shanghai, China).

### Processing of Sequencing Data

The 16S rRNA sequencing data were processed using the Quantitative Insights Into Microbial Ecology (QIIME) platform (V.1.9.1) (Kuczynski et al., 2011). The raw 16S rRNA reads were demultiplexed, quality filtered by Trimmomatic and merged by FLASH with the following criteria (Magoč and Salzberg, 2011): (1) the 300 bp reads were truncated at any site receiving an average quality score < 20 over a 50 bp sliding window, and the truncated reads shorter than 50 bp were discarded; (2) reads with exact barcode matching, 2 nucleotide mismatches in primers, or ambiguous characters were removed; and (3) only overlapping sequences longer than 10 bp were assembled according to their overlapping sequence. Reads that could not be assembled were discarded.

Operational taxonomic units (OTUs) were clustered with a 97% similarity cutoff using UPARSE (version 7.1)^2^, and chimeric sequences were identified and removed using UCHIME (Edgar et al., 2011; Edgar, 2013). The taxonomy of each representative OTU sequence was assigned by RDP Classifier^3^ via comparison with the 16S rRNA database using a confidence threshold of 0.7 (Navas-Molina et al., 2013).

### Imputed Metagenomic Analysis

The metagenomes of the gut microbiome were imputed from 16S rRNA sequences with Phylogenetic Investigation of Communities by Reconstruction of Unobserved States (PICRUSt) (Langille et al., 2013). This method predicts gene family abundance from phylogenetic information with an estimated accuracy of 0.8. The closed OTU table was used as the input for metagenome imputation and was first rarefied to a single sequencing depth prior to the PICRUSt analysis. Next, the resulting OTU table was normalized by 16S rRNA gene copy number. The gene content was predicted for each individual. Then, the predicted functional composition profiles were collapsed into Kyoto Encyclopedia of Genes and Genomes (KEEG) database pathways (Tang et al., 2018). Pathways present in < 10% of the samples were not included in the comparison analysis (Tang et al., 2018).

### Statistical Analysis

All statistical analyses were performed using R packages (V.2.15.3). For baseline characteristics, chi-square tests were used for dichotomous variables, and independent-samples *t*-tests were used for continuous variables. We performed Adonis analysis of body mass index (BMI), dietary habits, age, and gender, with statistical significance determined at an alpha level of 0.05. All alpha indexes were investigated using QIIME (Kuczynski et al., 2011). Statistical analysis of alpha diversity indexes between the two groups was performed with Student’s *t*-test. Then, the beta diversity index was calculated by weighted UniFrac distances. The linear discriminant analysis (LDA) effect size (LEfSe) method was used to analyze biomarker taxa for patient and healthy group detection (Segata et al., 2011). First, taxa with significant differential abundances were detected by a non-parametric factorial Kruskal-Wallis (KW) rank sum test. Second, a (unpaired) Wilcoxon rank sum test was employed to assess the biological consistency among subclasses. LDA was utilized to examine the effect size of each differentially abundant trait, and a strict threshold of 3.5 was selected for logarithmic LDA scores. Alpha values of 0.05 were used for the KW rank sum test.

The differences between specific taxa were determined using ANOVA (analysis of variance) for multiple testing and the Wilcoxon rank sum test for intergroup difference testing, with Benjamini-Hochberg false discovery rate (FDR) corrections. *P* < 0.05 were considered significant.

A random forest model using the 10-genus signature was applied to the data from the samples of patients and HCs and DRE vs. DSE, respectively. Operating characteristic curves (receiving operating curves, ROCs) were constructed, and the area under the curve (AUC) was calculated to determine the discriminatory ability of the random forest model (Wei et al., 2020).

## Results

### Demographics and Clinical Data

Fifty-five patients with epilepsy and 46 HCs were enrolled in this explorative cross-sectional study. Thirteen patients with the disease and 10 HCs were subsequently included in the validation cohort. There were no statistically significant differences between patients and HCs in clinical features which included age, gender, weight, height, BMI, blood pressure, or 27 dietary factors. All data in the exploration and validation cohort are summarized in Table 1.

**TABLE 1:** Demographic of the study populations.

	**Exploration cohort**			**Validation cohort**		
	**EP**	**HC**	***P*-value**	**EP**	**HC**	***P*-value**
No. of patient	55	46		13	10	
Sex (F/M)	28/27	24/22	0.94^a^	7/6	5/5	1^a^
Age (year)^d^	26.33 ± 12.05	28.5 ± 4.27	0.26^b^	32.96 ± 3.69	28.7 ± 2.15	0.54^b^
Weight (kg)^d^	55.06 ± 10.65	55.17 ± 6.62	0.97^b^	60.63 ± 9.65	63.24 ± 6.48	0.66^b^
Height (m)^d^	1.60 ± 0.11	1.63 ± 0.07	0.41^b^	1.65 ± 0.95	1.61 ± 0.36	0.87^b^
BMI (kg/m^2^)^d^	21.33 ± 2.72	20.76 ± 1.19	0.23^b^	22.01 ± 1.71	21.86 ± 2.12	0.82^b^
SBp (mmHg)^d^	117 ± 13.72	112 ± 8.55	0.64^b^	109 ± 9.11	119 ± 13.67	0.44^b^
DBp (mmHg)^d^	76.8 ± 11.13	75 ± 8.82	0.33^b^	71.2 ± 14.22	73 ± 6.72	0.07^b^
Age of onset (year)^d^	18.34 ± 11.89	0		14.17 ± 6.89	0	
**Medication duration**						
≤ 1 year	4	0		2	0	
>1 year	51	0		11	0	
**Seizure frequency**						
≤ 4/year	23	0		8	0	
>4/year	32	0		5	0	
**Medication**						
Valproate	32	0		2	0	
Levetiracetam	21	0		5	0	
Oxcarbazepine	16	0		3	0	
Lamotrigine	12	0		3	0	
Carbamazepine	8	0		1	0	
Topiramate	5	0		2	0	
Phenobarbital	1	0		0	0	
**Seizure type**						
Focal onset	4	0		2	0	
Generalized onset	36	0		9	0	
Unknown onset	15	0		2	0	
**Response to the drug**						
DRE	30	0		8	0	
DSE	25	0		5	0	
**Nutrients**						
Energy (kcal)^d^	2476.33 ± 51.31	2349.11 ± 44.37	0.48^c^	2297.33 ± 79.55	2322 ± 60.77	0.19^c^
Protein(g)^d^	100.26 ± 20.52	103.66 ± 9.03	0.68^c^	95.31 ± 11.07	90.22 ± 17.85	0.48^c^
Fat (g)^d^	48.72 ± 9.11	56.47 ± 14.09	0.71^c^	54.32 ± 6.63	53.15 ± 8.80	0.91^c^
Carbohydrate (g)^d^	386.00 ± 30.14	320.11 ± 40.97	0.06^c^	366.08 ± 24.33	334.46 ± 30.96	0.35^c^
Fiber (g)^d^	25.26 ± 4.31	24.92 ± 3.37	0.34^c^	24.22 ± 7.05	25.19 ± 7.73	0.40^c^
Cholesterol (mg)^d^	402.33 ± 77.03	490.66 ± 74.03	0.82^c^	446.51 ± 63.09	497.66 ± 90.03	0.30^c^
VitaminA (ug/RE)^d^	471.30 ± 113.51	535.57 ± 212.23	0.15^c^	488.31 ± 17.98	500.10 ± 30.66	0.08^c^
Thiamine (mg)^d^	1.83 ± 0.77	1.71 ± 1.99	0.15^c^	1.69 ± 0.47	1.55 ± 1.03	0.32^c^
Riboflavin (mg)^d^	1.14 ± 0.52	1.33 ± 0.21	0.89^c^	1.49 ± 0.24	1.20 ± 0.51	0.28^c^
Vitamin B6 (mg)^d^	0.47 ± 0.34	0.41 ± 0.35	0.51^c^	0.61 ± 0.12	0.41 ± 0.35	0.51^c^
Vitamin B12 (ug)^d^	0.44 ± 0.89	0.34 ± 0.91	0.43^c^	0.10 ± 0.01	0.34 ± 0.10	0.10^c^
Folvite (ug)^d^	450.80 ± 90.34	500.90 ± 106.14	0.06^c^	516.33 ± 77.12	600.07 ± 80.11	0.61^c^
Niacin (mg)^d^	16.14 ± 2.02	17.33 ± 4.40	0.87^c^	17.99 ± 2.60	16.71 ± 6.19	0.70^c^
Vitamin C (mg)^d^	201.90 ± 90.33	186.91 ± 73.12	0.44^c^	180.30 ± 40.93	178.66 ± 33.63	0.94^c^
Vitamin E (mg)^d^	34.05 ± 4.91	46.36 ± 10.11	0.63^c^	39.14 ± 4.69	44.01 ± 6.69	0.14^c^
Ca (mg)^d^	810.10 ± 92.21	1003.14 ± 80.40	0.67^c^	990.10 ± 63.43	1120.10 ± 96.88	0.17^c^
P (mg)^d^	1673.20 ± 49.79	1584.00 ± 94.74	0.73^c^	1573.21 ± 80.41	1584.00 ± 76.66	0.63^c^
K (mg)^d^	2182.71 ± 77.14	2536.14 ± 72.71	0.35^c^	2661.83 ± 82.29	2634.14 ± 92.71	0.10^c^
Na (mg)^d^	1576.88 ± 41.82	1799.31 ± 90.13	0.79^c^	1856.71 ± 82.41	1602.19 ± 40.61	0.09^c^
Mg (mg)^d^	399.47 ± 96.83	492.71 ± 71.99	0.17^c^	463.12 ± 74.55	500.31 ± 60.52	0.22^c^
Fe (mg)^d^	27.10 ± 8.66	25.10 ± 6.74	0.09^c^	26.06 ± 6.45	27.84 ± 1.47	0.43^c^
Zn (mg)^d^	17.33 ± 1.18	16.94 ± 1.00	0.39^c^	18.71 ± 0.99	16.62 ± 1.18	0.92^c^
Se (ug)^d^	74.33 ± 1.34	73.15 ± 2.71	0.53^c^	68.19 ± 6.55	70.01 ± 6.16	0.11^c^
Cu (mg)^d^	3.01 ± 1.96	1.95 ± 0.72	0.77^c^	3.36 ± 1.84	2.45 ± 1.32	0.47^c^
Mn (mg)^d^	8.35 ± 1.42	7.12 ± 1.93	0.51^c^	9.01 ± 0.39	8.14 ± 1.11	0.84^c^
I (mg)^d^	136.78 ± 19.41	141.95 ± 26.11	0.45^c^	128.29 ± 17.64	149.67 ± 26.74	0.64^c^
Vitamin D (ug)^d^	2.90 ± 0.84	2.62 ± 0.16	0.94^c^	2.71 ± 1.32	2.79 ± 0.70	0.42^c^

*^1^_χ_^2−^test. ^*b*^Student’s t-test. ^*c*^ANOVA was used to compare the means of each nutrient between the four groups and post hoc tests. ^*d*^Measurement data are expressed as the mean ± SD according to the normality of distribution. EP, patients with epilepsy; HC, healthy spouse-matched control; BMI, body mass index; SBp, systolic blood pressure; DBp, diastolic blood pressure; EEG, electroencephalogram; AEDs, antiepileptic drugs; DRE, drug-resistant epilepsy; DSE, drug-sensitive epilepsy.*

### Alteration in Sample Community Diversity and Configuration Between Patients With Epilepsy and HC Groups

**FIGURE 1:**
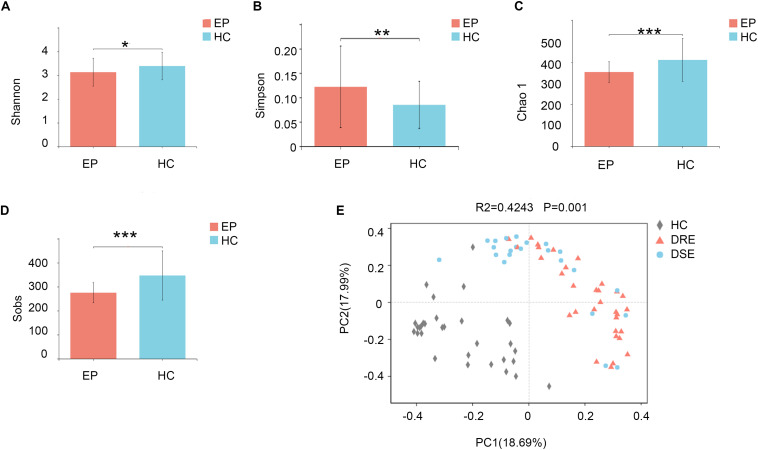
Comparison of fecal microbiota structure between patients with epilepsy and HCs. The **(A)** Shannon (*p* = 0.02*) and **(B)** Simpson (*p* = 0.009**) indexes were used to detect higher community diversity in terms of both richness and evenness in the patient group. The **(C)** Chao1 index (*p* = 0.0003***) revealed greater community richness. **(D)** The observed species (*p* = 0.0001***) were used to characterize good sequencing depths. The alpha diversity indexes revealed significantly decreased ecological diversity in the fecal microbiome in patients with epilepsy (*n* = 55) compared with individuals without epilepsy (*n* = 46). **(E)** PCoA plots based on the relative abundances of microbiota indicated that the microbiome samples were clustered by group (DRE, DSE, and HC) (PC1 = 18.69%, PC2 = 17.99%). Notes: *p < 0.05, 0.001 < *p* < 0.01**, *p* < 0.001***; EP, patients with epilepsy; HC, healthy spouse-matched controls; DRE, drug-resistant epilepsy; DSE, drug-sensitive epilepsy.

To profile the differences in fecal microbiota structure among participants, we performed Illumina MiSeq sequencing of the V3-V4 region of the bacterial 16S rRNA gene for 101 samples collected from the two groups in the exploration cohort. We observed significantly lower microbiota diversity in patients with epilepsy than in HCs. The Shannon (3.13 ± 0.58 vs. 3.39 ± 0.58, *p* = 0.0286) (Figure 1A) and Simpson (0.12 ± 0.08 vs. 0.08 ± 0.04, *p* = 0.0094) (Figure 1B) index results revealed statistically significant differences in species evenness and community diversity among study participants. The Chao1 index (353.82 ± 48.90 vs. 411.54 ± 102.13, *p* = 0.0003) (Figure 1C) was used to represent community richness, and the data suggested that participants with epilepsy exhibited lower species richness than HCs; the number of observed species (Sobs) (275.33 ± 41.64 vs. 347.26 ± 102.40, *p* = 0.0001) (Figure 1D) was used to reflect sequencing depth, which suggested that good coverage was achieved in the tests for all groups.

### Sample Structure Comparison Analysis Between Patients With Epilepsy and HC Groups and Within Subgroups With Different Clinical Phenotypes and Features

To examine the bacterial community structure in 101 fecal samples from patients with epilepsy and HCs, a non-parametric multivariate analysis of variance (Adonis) based on weighted UniFrac distances was performed, and the calculated *p*-values (*R*^2^ = 0.4243, *p* = 0.001 for Adonis) further demonstrated marked differences in the bacterial communities between the groups.

We performed principal coordinate analysis (PCoA) based on weighted UniFrac distances at the genus level for the patients with epilepsy with different drug responses. The overall structures of the bacterial communities in patients with DRE and DSE and HCs clustered separately (Figure 1E), which suggested that different clinical phenotypes and characteristics of patients with epilepsy have distinct profiles.

### Difference Analysis of Gut Microbiota Abundance in Patients With Epilepsy and Control Groups and Between Subgroups

**FIGURE 2:**
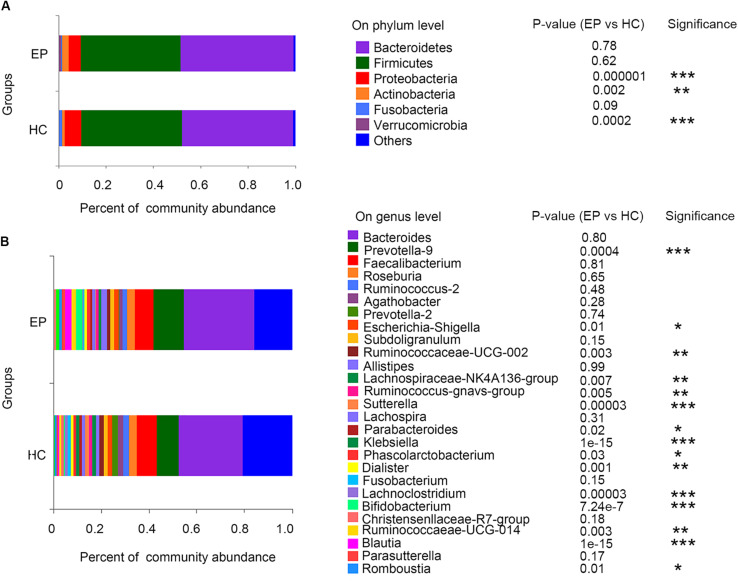
Relative abundance of fecal microbiome members in patient groups and the control group. **(A)** Variations in the relative abundances of the top 7 fecal bacterial taxa at the phylum level; **(B)** heatmap of the abundances of the top 30 fecal bacterial taxa at the genus level. EP, patients with epilepsy; HC, healthy spouse-matched controls.

In the disease and HC groups, the fecal microbiota exhibited a typical human diversity profile dominated by *Firmicutes*, *Bacteroidetes*, *Actinobacteria*, *Proteobacteria*, and other rare bacterial phyla (Figures 2A,B).

**FIGURE 3:**
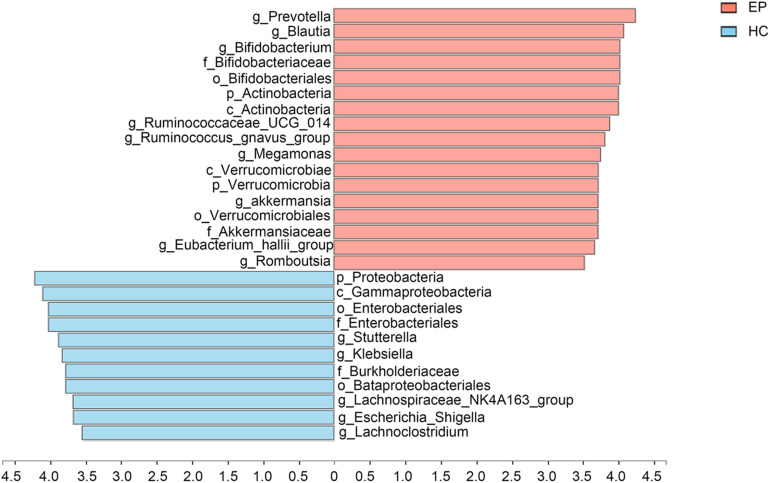
Marked differences in the abundance of fecal microbiome members at all taxonomic levels between EP and HC groups obtained by using the linear discriminant analysis (LDA) effect size (LEfSe) method; *p* < 0.05, LDA > 3.5. EP, patients with epilepsy; HC, healthy spouse-matched controls.

A total of 7,626,858,530 bases were obtained from all patients and HCs. After chimera exclusion and filtering, we acquired a total of 17,020,295 sequences from the patients and HCs. The search for presumed distinctive microbial biomarkers was performed by using the LEfSe method. LDA was employed to assess the effect size of every differentially abundant trait. We selected a strict threshold of 3.5 for logarithmic LDA scores. Alpha values of 0.05 were used for the KW rank sum test. We particularly focused on abundance at the level of genus and phylum (Figure 3). Our data showed that at the phylum level, *Actinobacteria* and *Verrucomicrobia* were significantly outnumbered in the disease group, while *Proteobacteria* were less abundant in the disease group. At the genus level, disease samples showed a higher abundance of *Prevotella_9*, *Blautia*, *Bifidobacterium*, *Ruminococcaceae_UCG_014*, *Ruminococcus__gnavus_group*, *Megamonas*, *Akkermansia*, *Eubacterium__hallii_group* and *Romboutsia* but a decreased abundance of *Sutterella*, *Klebsiella*, *Lachnospiraceae_NK4A136_group*, *Escherichia_Shigella*, and *Lachnoclostridium*.

**FIGURE 4:**
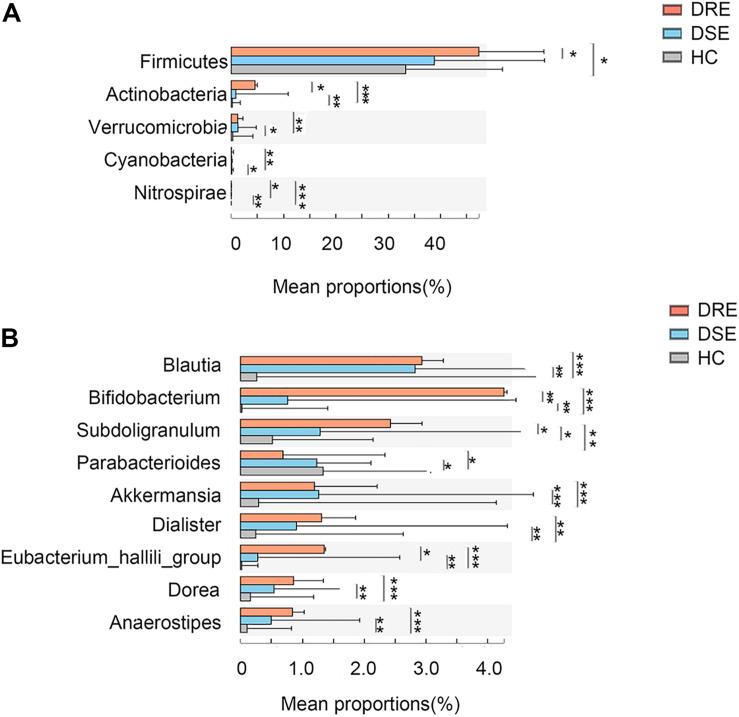
Microbiome alterations in patients with different clinical prognosis outcomes and HCs. The relative abundances of 5 taxa at the phylum **(A)** and 8 taxa at the genus **(B)** level were significantly different among three groups. (DRE: 30, red; DSE: 25, blue) and HCs (*n* = 46) after correcting for confounding variables. DRE, drug-resistant epilepsy; DSE, drug-sensitive epilepsy; HC, healthy spouse-matched controls. *p < 0.05, 0.001 < *p* < 0.01**, *p* < 0.001***.

Additionally, to observe whether any feature of the gut microbiota distinguished patients with different drug responses, we performed disease subgroup analysis by stratifying patients into those with DRE and DSE (Figures 4A,B). The phyla *Actinobacteria* (4.53% vs. 0.87% vs. 0.19% in the DRE, DSE, and HC groups, respectively, *p* = 0.0001), *Verrucomicrobia* (1.27% vs. 1.20% vs. 0.29%, *p* = 0.001) and *Nitrospirae* (0.003% vs. 0.002% vs. 0%, *p* = 0.00001) and the genera *Blautia* (2.93% vs. 2.82% vs. 0.26%, *p* = 0.0001), *Bifidobacterium* (4.25% vs. 0.75% vs. 0.02%, *p* = 0.057), *Subdoligranulum* (2.43% vs. 1.28% vs. 0.51%, *p* = 0.005), *Dialister* (1.30% vs. 0.91% vs. 0.24%, *p* = 0.001), and *Anaerostipes* (0.83% vs. 0.49% vs. 0.10%, *p* = 0.0001) were enriched in the DRE group in comparison with the DSE and HC groups. The phylum *Cyanobacteria* (0.03% vs. 0.07% vs. 0.09%, *p* = 0.001) and genus *Parabacteroides* (0.68% vs. 1.23% vs. 1.33%, *p* = 0.01) were significantly depleted in the DRE group.

### Disease Status Discrimination With the 10-Genera Microbiome Signature

**FIGURE 5:**
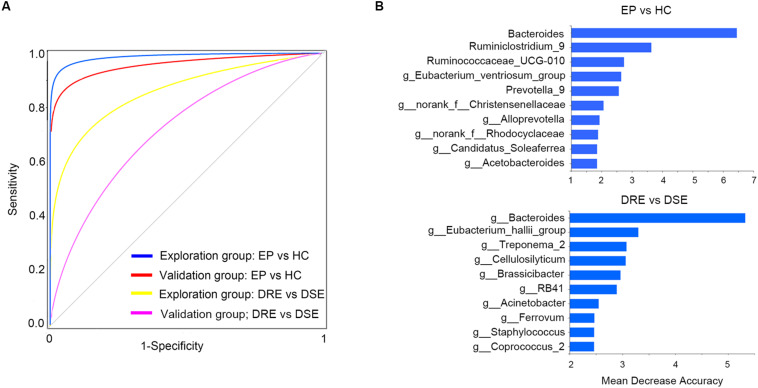
High diagnostic accuracy of a gut microbiome signature for the detection of epilepsy. **(A)** ROC analysis was used to assess predictive model performance for the exploration and validation cohorts. The AUC was 0.97 for the exploration cohort (disease: 55, control: 46, blue) and 0.96 for the validation cohort (disease: 13, control: 10, red). The AUC for discriminating the patients with DRE from those with DSE in exploration cohort was 0.85 (DRE: 30, DSE: 25, yellow) and 0.72 (DRE: 8, DSE: 5, purple). **(B)** The detailed explanatory variables based on the random forest model in each comparison. The lengths of the bars in the histogram indicate the mean decrease accuracy, which represent the importance of the gut microbiome module for classification. EP, patients with epilepsy; HC, healthy spouse-matched controls; DRE, drug-resistant epilepsy; DSE, drug-sensitive epilepsy.

To determine whether gut bacteria can be regarded as biomarkers for distinguishing different disease outcomes and a lack of disease, we constructed a random forest model based on a gut microbiome signature composed of the relative abundances of the top 10 disease associated genera effectively distinguished EP vs. HC, DRE vs. DSE samples, respectively. We established four models, namely, EP vs. HC in the exploration and validation cohorts and DRE vs. DSE in the exploration and validation cohort (Figure 5A).

The model performance was evaluated using ROC analysis, which indicated an AUC value of 0.97 in the exploration cohort (95% CI: 0.93–0.99). The sensitivity and specificity for distinguishing epilepsy patients from HCs were 98 and 100%, respectively. Next, we verified the discriminatory power of the model using an independent validation cohort containing 13 patients with epilepsy and 10 HCs and observed very good diagnostic accuracy by using the same biomarker taxa from exploration group (Figure 5B). The AUC was 0.95 (95% CI: 0.91–0.96) in the validation cohort. The sensitivity and specificity of the gut microbiota in segregating patients with epilepsy and HCs were 87 and 95%, respectively, which confirmed that the gut microbiome-based classifier could precisely distinguish epilepsy from a lack of epilepsy. Notably, by using the biomarker taxa to discriminate DRE vs. DSE (Figure 5B), the AUC for this comparison was 0.85 (95% CI: 0.69–0.94) in exploration group. We also obtained certain accuracy when classifying DSE from DSE in validation group, with AUC was 0.72 (95% CI: 0.60–0.83). These findings revealed that these models based on the fecal microbiota could distinguish between patients with epilepsy on the basis of different drug responses and between patients with epilepsy and healthy control in this western Chinese cohort, indicating that gut microbiota profile could be utilized in patient identification.

### Predicted Function Analysis of Microbiome in Patients With Epilepsy and HC

To verify further the involvement of the “gut microbiota-brain” axis in epilepsy, PICRUSt was utilized in the prediction of KEGG functional orthologs (Liu et al., 2019). Table 2 provides the broad functions potentially linking the gut microbiome and epilepsy, including metabolism, environmental information processing, genetic information processing, organism system and cellular process. In total, 30 functional orthologs were significantly different between patients and HCs when using the level-2 KEGG pathways. The orthologs enriched in patients with epilepsy included amino acid metabolism, carbohydrate metabolism, lipid metabolism, cellular signaling and processes, biosynthesis and metabolism of glycan, neurodegenerative diseases, and genetic information processing, among others. On the contrary, the most common markers in the HCs were related to secondary metabolites biosynthesis, cell proliferation and apoptosis, cell migration, energy metabolism, environmental adaptation, enzyme families, folding, sorting and degradation, immune system, metabolism of cofactors and vitamins, nervous system, nucleotide metabolism, DNA repair, replication, and translation, as well as transport and catabolism.

**TABLE 2:** Predicted COG functional pathway differences inferred from 16S rRNA gene sequences using PICRUSt.

		**EP% (mean ± SD)**	**HC% (mean ± SD)**	***p*-value**
EP enriched modules	Amino acid metabolism	11.54 ± 3.41	11.59 ± 3.44	0.041
	Carbohydrate metabolism	13.35 ± 3.95	12.90 ± 3.83	0.013
	Cellular processes and signaling	5.27 ± 1.56	5.04 ± 1.49	0.019
	Glycan biosynthesis and metabolism	3.16 ± 0.93	3.10 ± 0.91	0.002
	Lipid metabolism	3.44 ± 1.02	3.41 ± 0.01	0.004
	Metabolism	3.11 ± 0.92	2.91 ± 0.86	0.022
	Metabolism of other amino acids	1.81 ± 0.53	1.77 ± 0.52	0.006
	Metabolism of terpenoids and polyketides	1.99 ± 0.59	1.98 ± 0.58	0.048
	Neurodegenerative diseases	0.13 ± 0.03	0.10 ± 0.10	0.001
	Signal transduction	1.85 ± 0.54	1.74 ± 0.51	0.002
	Signaling molecules and interaction	0.22 ± 0.06	0.21 ± 0.06	0.016
	Xenobiotics biodegradation and metabolism	2.11 ± 0.62	1.82 ± 0.54	0.033
	Immune system diseases	0.06 ± 0.01	0.05 ± 0.01	0.054
	Genetic information processing	3.13 ± 0.92	3.12 ± 0.92	0.002
HC enriched modules	Biosynthesis of other secondary metabolites	1.14 ± 0.33	1.21 ± 0.35	0.012
	Cell growth and death	0.59 ± 0.17	0.61 ± 0.18	0.003
	Cell motility	1.63 ± 0.48	2.19 ± 0.65	0.045
	Energy metabolism	7.00 ± 2.20	7.11 ± 2.11	0.001
	Environmental adaptation	0.17 ± 0.05	0.19 ± 0.05	0.036
	Enzyme families	2.63 ± 0.77	2.63 ± 0.78	0.037
	Folding, sorting and degradation	3.00 ± 0.89	3.06 ± 0.91	0.018
	Immune system	0.10 ± 0.03	0.11 ± 0.03	0.003
	Metabolism of cofactors and vitamins	5.21 ± 1.54	5.32 ± 1.58	0.012
	Nervous system	0.13 ± 0.03	0.13 ± 0.04	0.001
	Nucleotide metabolism	4.86 ± 1.44	4.89 ± 1.45	0.033
	Replication and repair	10.63 ± 3.14	10.88 ± 3.22	0.026
	Transcription	3.48 ± 1.03	3.35 ± 0.99	0.026
	Translation	6.53 ± 1.93	6.83 ± 2.02	0.001
	Transport and catabolism	0.39 ± 0.11	0.41 ± 0.12	0.002

*EP, patients with epilepsy; HC, healthy spouse-matched control; PICRUSt, Phylogenetic Investigation of Communities by Reconstruction of Unobserved States; KEGG, Kyoto Encyclopedia of Genes and Genomes; SD: standard deviation.*

## Discussion

In recent years, the role of the gut microbiota-brain axis in the pathogenesis of central nervous system (CNS) disease has received increasing attention. However, the impact of the fecal microbiota on epilepsy is poorly understood. Recent experimental data revealed that human intestinal microbes play a vital role in host body defense against pathogenesis (Guarner and Malagelada, 2003; Ishaq et al., 2018; Laing et al., 2018). Hence, 16S rRNA sequencing with the MiSeq platform was used to assess the bacterial community in patients with epilepsy. To increase the reliability of our results, we described the microbial communities related to epilepsy in details, particularly with regards to a clinical setting. Also, we employed prediction models to identify differentially abundant bacterial taxa for disease diagnosis (Qian et al., 2018). The recruitment of healthy spouses and strict diet control were additional important advantages that could have, to a large extent, mitigated the effect of diet and the environment on the outcomes.

Our findings indicated that the alpha diversity indexes of the disease group were much lower than those of the household control group. Relatively lower microbial diversity has recently been linked to drug refractory epilepsy in children (Lindefeldt et al., 2019) and other conditions linked to CNS alterations, including Alzheimer’s disease (Liu et al., 2019), multiple sclerosis (Jangi et al., 2016), and Parkinson’s disease (Hill-Burns et al., 2017).

In addition, unfavorable prognosis is more common in patients with epilepsy if their epilepsy is drug resistant (Devinsky et al., 2018). All these conditions are associated with increased risks of injury and death, a greater medication burden, increased adverse effects and increased comorbidities (Devinsky et al., 2018). Therefore, subgroup analyses were also conducted in our study, and the results of PCoA suggested that the fecal microbiota profile different significantly between patients with DRE and DSE and HCs.

Our results showed that some phyla, including *Fusobacteria*, *Verrucomicrobia*, and *Nitrospirae*, were overgrown in the disease group and that *Firmicutes* and *Saccharibacteria* were less abundant in the disease group. Existing studies have indicated that *Fusobacteria* is pathogenic to vertebrates and prevalent in both human colorectal carcinoma and inflamed gut mucosa (Roggenbuck et al., 2014). Some investigators described *Fusobacterium* species as pathobionts given their invasive nature and ability to translocate to the blood and contribute to systemic disease states (Volkmann, 2017; Yang et al., 2017). The phylum *Verrucomicrobia* is known for high abundances of short-chain fatty acid (SCFA)-producing and mucin-degrading microbes (de la Cuesta-Zuluaga et al., 2017). *Verrucomicrobia* can degrade mucin, which might disturb the integrity of the intestinal barrier and subsequent bacterial translocation. *Nitrospirae* can increase nitrite toxicity, which might ultimately cause blood-brain barrier dysfunction and permeability and contribute to the mechanism of action in epilepsy. *Saccharibacteria* are much lower in abundance in patients with some chronic inflammatory diseases than in healthy individuals (Cao et al., 2018), which suggests that *Saccharibacteria* play a protective role in immune defense.

Evidence has shown that the main function of *Prevotella copri* is to promote inflammation (Pedersen et al., 2016). Higher abundances of *Prevotella copri* could lead to the continuous production of IL-6 in the gut, which could trigger an inflammatory response (Leite et al., 2017). Moreover, *Prevotella* species reportedly alter gut permeability (Pedersen et al., 2016). The genera *Blautia*, *Bifidobacterium*, *Subdoligranulum*, *Dialister*, and *Anaerostipes* increased in patients with a poor prognosis. *Blautia* and *Subdoligranulum* are known for their SCFA-producing lineages (Peng et al., 2009), such as butyrate producers. Some studies have indicated that these small molecules can affect the barrier function of the gut and exert immune-regulatory effects (Maslowski et al., 2009), which means that SCFAs might influence epilepsy. The significant increase in *Bifidobacterium* in patients with epilepsy observed herein is in agreement with findings from two published studies for patients with refractory epilepsy (Lindefeldt et al., 2019; Ang et al., 2020). *In vivo*, as well as *in vitro* studies have implicated ketone bodies in the selective inhibition of *Bifidobacterial* growth and reduction of Th17 cells (intestinal pro-inflammatory cells) to produce anti-seizure effect (Olson et al., 2020). In addition, functional analysis in our study also revealed a significantly increased carbohydrate metabolism in disease group. It is established that the disturbance of carbohydrate metabolites may play a potential role in mechanism of epileptogenesis (Wang et al., 2016). *Bifidobacterium* digest complex carbohydrates and exhibit one of the largest predicted glycobiomes (Turroni et al., 2018; Lindefeldt et al., 2019). This pathway is superior with regards to the energy output produced by pathways utilized by other fermentative gut bacteria (Lindefeldt et al., 2019). Using predicted functional profile these alterations could explain concomitant increased proportion of *Bifidobacterium* and genes that facilitate the metabolism of carbohydrates in patients with epilepsy.

Epilepsy-associated microbial dysbiosis was characterized by changes in the abundances of some genera. The combination of the top 10 taxa associated with epilepsy on the basis of altered abundance discriminated patients with epilepsy from HCs with high accuracy. To evaluate which taxa were related to epilepsy and thus may serve as potential biomarkers for epilepsy, an independent cohort composed of patients (*N* = 13) and controls (*N* = 10) was used to validate the results. Subsequently, we also obtained good results in the validation cohort. This confirms that the gut microbiota can be used in accurate patient identification. Although this represents a new method for the diagnosis of epilepsy, large samples are needed to verify its accuracy.

Nonetheless, our study has certain limitations. First, it was not possible to directly link gut microbiome to the etiology of epilepsy, given that this is an association study, from which one is unable to draw a causal relationship. Second, 16S rRNA sequencing was not adequate to comprehensively reveal all possible factors that might have influenced disease status at the species or strain level (Caussy et al., 2019). We performed pathway analysis based on the metagenome obtained from 16S rRNA sequences. Even though metagenome inference approaches (PICRUSt) are widely applied in 16S rRNA studies, the application of shotgun metagenomic, metabolomic and metatranscriptomic technologies may reveal slight network interference in the genes of fecal microbes, regarding their expression, as well as the presence of microbial metabolites. This could expose the mechanism underlying disease development and thus complement taxonomic and phylogenetic data. Therefore, future research should consider the functional effects of changes in the gut microbiota on disease initiation, aggravation and progression, which will reveal how the complex “gut-brain-axis” facilitate the development of epilepsy.

## Data Availability Statement

The datasets generated for this study can be found in the raw reads were deposited into the NCBI Sequence Read Archive (SRA) database (Accession Number: SRP 272993).

## Ethics Statement

The studies involving human participants were reviewed and approved by the Research Ethics Committee of the Medical School of Sichuan University. The patients/participants provided their written informed consent to participate in this study.

## Author Contributions

XG designed and conceptualized study, analyzed the data, drafted the manuscript for intellectual content of the manuscript, carried out the statistical analysis, and interpreted the data. XL, CC, DZ, and DA collected fecal samples. ZH conceptualized and designed the study and revised the manuscript. All authors contributed to the article and approved the submitted version.

## Conflict of Interest

The authors declare that the research was conducted in the absence of any commercial or financial relationships that could be construed as a potential conflict of interest.

## Footnotes

1 http://db.foodmate.net/yingyangsu/

2 http://drive5.com/uparse/

3 http://rdp.cme.msu.edu/
